# The Response of microRNAs to Solar UVR in Skin-Resident Melanocytes Differs between Melanoma Patients and Healthy Persons

**DOI:** 10.1371/journal.pone.0154915

**Published:** 2016-05-05

**Authors:** Jingfeng Sha, Brian R. Gastman, Nathan Morris, Natasha A. Mesinkovska, Elma D. Baron, Kevin D. Cooper, Thomas McCormick, Joshua Arbesman, Marian L. Harter

**Affiliations:** 1 Department of Biochemistry, School of Medicine, Case Western Reserve University, Cleveland, OH, 44106, United States of America; 2 Department of Immunology, Cleveland Clinic, Cleveland, OH, 44195, United States of America; 3 Statistical Science Core in the Center for Clinical Investigation, Case Western Reserve University, Cleveland, OH, 44106, United States of America; 4 Department of Dermatology, Cleveland Clinic, Cleveland, OH, 44195, United States of America; 5 Department of Dermatology, Case Western Reserve University/University Hospitals Case Medical Center, Cleveland, OH, 44106, United States of America; 6 Case Comprehensive Cancer Center, School of Medicine, Case Western Reserve University, Cleveland, OH, 44106, United States of America; National Institutes of Health, UNITED STATES

## Abstract

The conversion of melanocytes into cutaneous melanoma is largely dictated by the effects of solar ultraviolet radiation (UVR). Yet to be described, however, is exactly how these cells are affected by intense solar UVR while residing in their natural microenvironment, and whether their response differs in persons with a history of melanoma when compared to that of healthy individuals. By using laser capture microdissection (LCM) to isolate a pure population of melanocytes from a small area of skin that had been intermittingly exposed or un-exposed to physiological doses of solar UVR, we can now report for the first time that the majority of UV-responsive microRNAs (miRNAs) in the melanocytes of a group of women with a history of melanoma are down-regulated when compared to those in the melanocytes of healthy controls. Among the miRNAs that were commonly and significantly down-regulated in each of these women were miR-193b (*P*<0.003), miR-342-3p (*P*<0.003), miR186 (*P*<0.007), miR-130a (*P*<0.007), and miR-146a (*P*<0.007). To identify genes potentially released from inhibition by these repressed UV-miRNAs, we analyzed databases (e.g., DIANA-TarBase) containing experimentally validated microRNA-gene interactions. In the end, this enabled us to construct UV-miRNA-gene regulatory networks consisting of individual genes with a probable gain-of-function being intersected not by one, but by several down-regulated UV-miRNAs. Most striking, however, was that these networks typified well-known regulatory modules involved in controlling the epithelial-to-mesenchymal transition and processes associated with the regulation of immune-evasion. We speculate that these pathways become activated by UVR resulting in miRNA down regulation only in melanocytes susceptible to melanoma, and that these changes could be partially responsible for empowering these cells toward tumor progression.

## Introduction

Cutaneous melanoma arises from the malignancy of melanocytes, a specialized cell that is responsible for the pigmentation and photo protection of human skin. This form of skin cancer is historically one of the most aggressive and drug-resistant of human cancers, and its incidence is increasing faster than any other cancer in the United States [1]. In fact, it has been estimated that ~ 76,000 new cases and 9700 deaths were expected to occur in 2014 [2], underscoring therefore the importance of developing new treatments for this disease.

Major challenges still exist in understanding how melanocytes are progressively converted into melanoma. At the heart of this challenge is the highly recognized link between intense, intermittent UVR exposure and melanoma. Recent progress has established a direct mutagenic role for UVR in the pathogenesis of this disease, as evidenced by the discovery of an elevated number of C > T (by UVB) or G > T (by UVA) transitions throughout a melanoma genome and in genes associated with recurrence [3–5]. Uses of mouse models for UVR-induced melanoma have also recently revealed that UVR-induced inflammation can promote melanocytic cell survival, immune-evasion [6], and the metastatic dissemination of melanoma cells [7]. Although these new studies underscore the importance of UVR’s contribution to the development of melanoma, the molecular mechanisms by which UVR permanently alters the homeostasis of a melanocyte that may be prone to malignant transformation remain unknown.

MicroRNAs help to restore cellular processes in response to external perturbations such as UVR [8, 9], and they have also been strongly implicated in the development and progression of many types of cancer, including melanoma [10]. Such small non-coding RNAs (~22-nucleotides), with a half-life of ~ 5 days, function primarily by inhibiting the translation and stability of mRNAs [8, 11]. Depending then on whether a miRNA is up-regulated or down-regulated, affected proteins in a shared pathway or in a signaling cascade may be respectively reduced or increased in concentration [8]. Consequently, miRNAs can regulate, either individually or collectively, the signal outputs of almost all cellular pathways, including those involved in differentiation, proliferation, inflammation, DNA repair, apoptosis, and the epithelial-to-mesenchymal transition [12].

Our current understanding of the effects of UVR on human melanocytes is mostly based on *in vitro* cell culture studies. In these types of studies, however, the melanocytes are no longer in their natural microenvironment, and therefore their response to UVR, as well as the functioning of their miRNAs, may not be faithfully represented. Thus, to obtain a more accurate portrait of the effects of solar UVR on the melanocytes in this context, we employed the technique of Laser Capture Microdissection (LCM). This technique enabled melanocytes from the skin of human volunteers to be obtained following their exposure to simulated UVR (ssUVR), that is the UVA and UVB rays of sunlight. This novel approach has enabled us to uncover new evidence suggesting that UVR can differentially affect the circuitry of miRNAs in the melanocytes of persons with a history of melanoma when compared to those of healthy individuals.

## Materials and Methods

### Study participants

Eight healthy fair-skinned females between the ages of 31 and 38, and with Fitzpatrick skin types of I or II [13], were enrolled in this study. None of them had a medical and/or dermatologic history, and each was closely matched in their exposure to sunlight over the years, particularly to the shoulder area. Additional exclusion criteria included smoking, pregnancy, and recreational sunbathing within 3 months of the start study.

Nine fair-skinned females with a history of having only one primary melanoma, with Fitzpatrick skin types of I or II [13], and between the ages of 35 to 46, were enrolled through a Melanoma Clinic at the Cleveland Clinic Foundation and the Case Western Reserve University/University Hospitals of Cleveland. Clinicopathologic information on each of these volunteers is reviewed in S1 Table.

Written informed consent was obtained from all of the persons who participated in the study, and all of the procedures described herein were approved by the University Hospital Case Medical Center Institutional Review Board (IRB), Case Western Reserve University.

### UV light source and MED determination

Simulated solar ultraviolet radiation (ssUVR) was delivered using a 1000 W xenon arc solar simulator model 81291 (Oriel Instruments, Stratford, CT), with a dichroic mirror and 81017bis filter (WG320/1.5 mm). This instrument importantly produces a spectral output similar to natural sunlight (290–400 nm). Lamp output was measured before each irradiation using an IL1700 radiometer (International Light, Newburyport, MA) equipped with a sensor for UVA (SED 033, UVA filter 19672) and UVB (SED 240, UVB filter 15541) and at 10 inches from the light source.

The MED, defined as the minimal dose of ssUVR required to produce a barely perceptible skin reddening (Erythema) after 24 h, was determined by exposing eight 1 cm areas on the posterior shoulder of each volunteer to increasing doses of ssUVR (~1 to 8 J per cm^2^ of total UV dose). Erythema was quantitatively assessed both visually and by colorimetric measurement using a chromometer (CR-300 Minolta, Tokyo, Japan). Linear regression was applied and 1 MED was calculated according to COLIPA recommendations [14] as the dose of UV producing an increase in the redness parameter (δα) of + 2.5. Based on this method, the average UV dose to induce 1 MED in the group of healthy volunteers and melanoma patients was 43.4 mJ/cm^2^ (~4.5 SED) and 112 mJ/cm^2^ (~4.1 SED), respectively. The MED data was converted to standard erythema dose (SED) values by using the CIE erythema action spectrum [6].

### *In vivo* ssUVR irradiation protocol

An area (6 mm circle) on the posterior shoulder of each volunteer received an ssUVR (UVA+UVB) dose of 4.0 times their baseline MED, and after 24 h, this spot was again exposed to the same amount of ssUVR. This level of exposure approximates 16 to 18 SEDs of UVR during each session. The following day (24 h later), the irradiated site, along with an adjacent non-exposed site, was removed by using a skin biopsy punch. Afterwards, the irradiated and un-irradiated tissues were immediately snap frozen in Tissue-Tek Optimal Cutting Temperature (OCT) embedding medium, and stored at -80°C until further use.

### Cryosectioning and immunohistochemical staining

Frozen tissue was sectioned into 8-mm slices at -24°C by the use of a cryostat. Sections and adjacent serial sections were mounted onto a Metal-Framed PEN membrane and glass microscopic slides, respectively, then stored at -80°C until further processing.

Monoclonal anti-human Melan-A/MART-1 (clone A103) and anti-CD68 antibodies (Dako) were used to identify melanocytes and macrophages in the tissue sections, respectively. To identify melanocytes in tissue sections for capture, the PEN membrane slides were fixed in cold acetone for 1 minute at 4°C, air dried, and then rehydrated in PBS. The slides were incubated with Melan-A antibody (dilution 1:25) along with an RNase inhibitor (Hoffman-La Roche) for 5 minutes. Afterwards, the slides were washed in PBS, rinsed in 3% H_2_0_2_, and washed again in PBS. The antigen, MART-1, a melanocyte specific protein [15], was detected by incubating the PEN membrane slides with a biotinylated conjugated secondary antibody and then with streptavidin-horseradish peroxidase (Dako’s LSAB2 System, HRP), for a total of 10 minutes. Staining was completed after incubating the slides for 5 minutes with 3–3’ diaminobenzidine (DAB) Substrate-Chromogen, which results in a brown-colored precipitate at the antigen site. Before commencing LCM, the stained PEN membrane slides were immediately dehydrated through increasing percentages of ethanol followed by xylene.

To identify potential macrophages, adjacent serial sections on glass slides were stained with antibodies to MART-1 (Melan-A, dilution 1:25) and CD68 (dilution 1:100). All antigens were detected by using Dako’s LSAB2 System, HRP, and (DAB) Substrate-Chromogen. The slides were viewed using the Arcturus Veritas laser Microdissection microscope at a magnification of 20X.

### Laser capture microdissection (LCM)

Arcturus^XTTM^ LCM System with a Nikon Eclipse Ti-E Microscope Base (Life Technologies, Bedford, MA) was used to capture individual melanocytes or a group of keratinocytes under 40X magnification. The instrument has two lasers, one of which is an infrared (IR) laser, and the other a UV laser. Given the structural characteristics of human skin, both lasers were used to capture the melanocytes, and with empirically determined parameters to maintain not only their bimolecular integrity, but also to minimize the presence of contaminating keratinocytes. The settings for the IR laser were as follows: 51 to 57 mW in power, and 10 to 15 ms in pulse duration. The UV laser was used at a cutting focus of 15% and a cutting speed of 500 μm per second. On average, we captured 500–700 of the irradiated or un-irradiated melanocytes per slide, although the actual yield is slightly lower since a single “spot” cutting by the LCM captures at best ~70–80% of an individual cell [16]

### Evaluating the purity of melanocytes after LCM

In a tissue section, ~200 irradiated melanocytes or keratinocytes were captured by LCM. Total RNA was isolated from each of these two types of cells using a single cell RNA and RNase-free DNase kit (Norgen Biotek Corp.). Total RNA (5 μl) was then subject to amplification using the Ovation PicoSL WTA System V2 (NuGEN Technologies, Inc.), and the cDNA purified by using QIAGENs’ MinElute Reaction Cleanup Kit. Concentration of the purified cDNA was measured by using the NanoDrop 2000 Spectrophotometer (Thermo Fisher Scientific, Inc.).

The TaqMan Gene Expression Assay system (Life Technologies) was used for quantitating transcriptional levels of the following genes: *MITF* (Hs01117294_m1), *MLANA (MART1)* (Hs00194133_m1), *KRT10* (Hs01043114_g1), and *KRT14* (Hs00265033_m1). The 18s rRNA gene (Hs03003631_g1) was used as an endogenous control, and, an RNA sample for the expression of a selected gene was run in triplicate along with a no-template-control. PCR reactions were performed on BIO-RADs’ CFX96 Real-Time System in 20 μl volumes at 95°C for 10 min, followed by 40 cycles of 95°C for 15 sec and 60°C for 1 min. Relative quantitation of mRNA expression was calculated by the 2^-ΔCt^ method [17]

### RNA extraction for microRNA profiling and Taqman miRNA low density arrays (TLDA)

Total RNA was extracted from the microdissected melanocytes using the RNAqueous-Micro RNA isolation Kit (Life Technologies). Briefly, cells were solubilized in 100 μl of lysis buffer, centrifuged, and incubated at 42°C for 30 min. Afterwards, 3 μl of isolation additive was added to the sample, followed by the addition of 1.25 volumes of 100% ethanol. The mixture was then passed through a microfilter cartridge assembly (Life Technologies) and subsequently washed according to the manufacturer-supplied procedure. Total RNA was then eluted from the column by using 20 μl of preheated (95°C) nuclease free water and then concentrated by vacuum centrifugation to a volume of 6 μl.

Total RNA (3 μl) was reverse transcribed by using a TaqMan microRNA reverse transcription kit (Life Technologies) with a stem-loop Megaplex primer pools (Human pools Set v2.0). These novel primers enable reverse transcription of mature miRNAs and endogenous controls. Briefly, and according to the instructions provided by Life Technologies, 3 μl of total RNA was supplemented with 0.8 μl of either pool A or pool B RT primer mix (10x), 0.8 μl RT buffer (10x), 1.5 μl of MultiScribe Reverse Transcriptase (50 U/ml), 0.2 μl of dNTPs with dTTP (100 mM), 0.9 μl of MgCl_2_ (25 mM), and 0.1 μl of RNAse inhibitor (20 U/μl) in a total reaction volume of 7.5 ml. A pulsed RT reaction was used to increase reverse transcription efficiency and entailed 40 cycles of 16°C for 2 min, 42°C for 1 min and 50°C for 1 sec, followed by a final reverse transcriptase inactivation at 85°C for 5 min.

The Megaplex RT product (2.5 μl) was pre-amplified using Life Technologies’ TaqMan PreAmp Master Mix (2x) and its Megaplex PreAMP primer pool A or B in a 25 μl PCR reaction. The pre-amplification cycling conditions were as follows: 95°C for 10 min, 55°C for 2 min, 72°C for 2 min followed by 18 cycles of 95°C for 15 s, 60°C for 4 min, and then 99.9°C for 10 min. This method has been previously shown to be highly sensitive and with unbiased amplification [18].

Applied Biosystems (ABI) TaqMan human microRNA arrays v2.0, consisting of cards A and B, were used for profiling the miRNAs in the irradiated and un-irradiated samples by qPCR. Card A, which focuses on more highly characterized miRNAs, contains 377 assays, while card B, which focuses on miRNAs that are rare and tissue specific, contain 290 assays. The TLDAs also include six candidate endogenous controls (e.g. MammuU6, RNU48, and RNU24) for correcting differences in the levels of RNA between samples. In our studies, the small nuclear RNA mammalian U6 (Mammu6), which is repeated four times on each card, was assigned as the endogenous control because of its stability and unresponsiveness to ssUVR (see S3 Fig).

### TLDA and data analysis

A reaction mixture consisting of 450 μl of TaqMan* Universal PCR Master Mix-No AmpErase* UNG (2x), 441 μl of nuclease-free water, and 9 μl of diluted PreAmp product (Pool A or B) was loaded onto a TLDA plate, which was then run in ABI’s 7900 HT Sequence Detection system. Raw C_t_ values were obtained by using its ABI’s TaqMan SDS v2.4 software. These values were then imported into ABI’s RQ manager or Expression Suite software to quantify the relative expression of miRNAs in each of the un-irradiated and irradiated samples. Both software packages use the comparative C_T_ method (ΔΔC_T_) [17], and when required, the baseline and threshold (C_T_) values of the assays were set manually according to ABI’s instructions. MicroRNAs with a C_T_ value >36 or which failed to amplify with an efficiency coefficient less than 0.65 were omitted from the analysis.

### Validation of TLDA results by qPCR

Results of the TLDA analysis were validated in two ways by using individual predesigned stem-loop qRT-PCR Taqman assays (Life Technologies) for miR-17-ID 002308, miR-25-ID 000403, miR-30c-ID 000419, miR-146a-ID 000468, miR-146b-5p-ID 001097, miR-199a-3p-ID 002304, miR-345-ID 002186, miR-106b-ID 000442, miR-151-3p-ID 002254, miR-186-ID 002285, miR-31-ID 002279, miR-106a-ID 002169, miR-149-ID 002255, and miR-331-3p-ID 000545. First, approximately 3000 irradiated or un-irradiated melanocytes were captured by LCM from the same tissue samples that were used in the microarray study. Total RNA was isolated from the captured melanocytes using RNAqueous-Micro RNA isolation kit (Life Technologies) or Single Cell RNA Purification kit (Norgen Biotek Corporation), and afterwards, a volume of ~ 15 μl was reversed transcribed to cDNA with the TaqMan RT kit (Life Technologies). qRT-PCR was performed using TaqMan Universal PCR Master Mix on an Applied Biosystems 7900 HT Fast Real-Time PCR system. Reactions were performed in duplicate, and in the end, microRNA expression was quantified by the comparative 2^-ΔΔCt^ method, normalizing Ct values to MammU6. Second, Taqman-based qRT-PCR was carried out on the remaining PreAmp products that were used in the microarray studies, and in triplicate.

### Assessment of ssUVR on MammU6 expression

To determine whether U6 snRNA (MammU6) abundance is altered by ssUVR, we used a melanocyte culture assay. Early passage (p3) normal human melanocytes from newborn foreskins (Specimen Resource Core, Yale University, New Haven, Ct) were cultured [9], and used to assess the effect of ssUVR on MammU6 expression and the DNA damage and repair genes, *GADD45a* and *XPC*, respectively. To acquire a quiescent state prior to ssUVR, the melanocytes were split into four plates and grown in medium containing 0.05% FBS for 24 h. Afterwards, three of these plates, covered with phosphate-buffered-saline (PBS), were irradiated with doses of either 5, 20, or 60 mJ/cm^2^, using the Oriel solar simulator (model 81291). As a control, the fourth plate was not subject to irradiation. After incubating for 24 h in complete medium, each plate was again exposed to the same ssUVR dosage. After the plates were returned to complete medium, they were incubated for another 24 h, and then harvested for total RNA isolation using the mirVana miRNA isolation kit (Life Technologies). We found no visual evidence of cell death or toxicity with these selected ssUVR doses.

The quality and quantity of the isolated RNA was assessed using the NanoDrop 2000 Spectrophotometer (Thermo Fisher Scientific, Inc), and afterwards, different concentrations of RNA, ranging from 1 to 80 ng from each of the un-irradiated or irradiated samples, was reversed transcribed using a TaqMan microRNA reverse transcription kit with a stem-loop MammU6 primer (Life Technologies). qPCR was then performed using a MammU6 TaqMan MicroRNA assay according to the manufacturer’s instructions (Life Technologies).

To validate the effect of ssUVR on the melanocytes, the RNA (200 ng) from each of the irradiated or un-irradiated melanocyte cultures was DNase treated using DNasen I (Life Technologies) and then reversed transcribed using Life Technologies’ SuperScript VILO MasterMix. The TaqMan Gene Expression Assay system (Life Technologies) was used for quantitating the transcriptional levels of the following genes: *GADD45A* (Hs00169255_m1) and *XPC* (Hs01104206_m1). The *GAPDH* gene (Hs02758991_g1) was used as an endogenous control, and an RNA sample for the expression of a selected gene was run in triplicate along with a no-template-control. PCR reactions were performed on BIO-RADs’ CFX96 Real-Time System according to the manufacturer’s instructions. Relative quantitation of mRNA expression in the irradiated and un-irradiated melanocytes was calculated by the 2^-ΔΔCt^ method [17]

To measure quiescence, a 1:100 dilution of BrdU labeling reagent (Life Technologies) was added to the culture for 1.5 h. Afterward, the cells were fixed in methanol for 20 min at -20°C, air dried, rehydrated with PBS, incubated with 3N HCl for 10 min, washed in PBS, and then incubated with 0.1M sodium borate for 10 min. The primary antibody used for immuno-fluorescence was the anti-BrdU monoclonal antibody conjugated to fluorescein isothiocyanate (FITC). Immunostaining of cell populations (~ 465 were counted) was examined using an Olympus IX71 microscope and Olympus TH4-100 Camera. The percentage of BrdU-positive cells was approximately 14% (data not shown).

### Predicted targets of miRNAs and IPA analysis

The “MicroRNA Target Filter” program of QIAGEN’s Ingenuity Pathway Analysis (IPA), www.ingenuity.com), and DIANA-TarBase v7.0 were used to identify the mRNA targets of the differentially expressed UV-responsive miRNAs listed in the text [19]. As a database, DIANA-TarBase holds more than 500,000 miRNA-target interactions, which have been validated experimentally. The IPA program uses TargetScan v6.2, which predicts with confidence (high or moderate) miRNA to mRNA targeting and the DIANA- TarBase v7.0 [19], as well. The scoring algorithm of TargetScan for predicting miRNA targeting takes two or more factors into account, one of which is the total context score [20]. For example, a total context score of -0.4, which corresponds to 2^−0.85^-fold change, indicates that the miRNA is predicted to repress the expression of its mRNA target to 55% of the “normal” level. For the most part, the analysis was run using IPA’s tools (Connect, Grow, Path Explorer, and Overlay) and outcomes were ranked according to statistical significance. Filters to prioritize mRNA targets included only direct relationships, all molecules, species: human (with relaxed filter), and relationship types such as RNA-RNA interactions (microRNA targets) or expression and transcription. Notably, the clustering of miRNAs in IPA resulted in the UV-responsive miRNAs miR-106a, miR-106b, miR-17, and miR-20a showing up as miR-17-5p; miR-19a and -19b as miR-19b-3p; miR-29a and 29c as miR-29a-3p and miR-29c-3p; miR-30b, -30c, and -30e as miR-30b-5p, miR-30c-5p, and miR-30e-5p; miR-195 and miR-16 as miR-16-5p; miR-106a and miR-20a as miR-17-5p; and miR-7 as miR-7-5p.

### Statistical analysis

The ΔΔC_T_ values generated by the Expression Suite Software were imported into R. For each sub-analysis, miRNAs with more than two missing values were filtered out. The statistical significance of differences in miRNA expression between the irradiated and un-irradiated melanocytes was determined by using a one-sample Wilcoxon signed-rank test on the log2-transformed RQ values. A Mann–Whitney test was also performed for comparing the differences between the healthy individuals and the melanoma patients relative to miRNA expression. The relationship between the C_T_ values of MammU6 and increasing doses of ssUVR was investigated by linear regression and multiple linear regression using C_T_ as the outcome and RNA and ssUVR as predictors. A *p*-value < 0.05 was regarded as statistically significant throughout the study. However for each set of analyses, we also report the *q*-value to control the false discovery rate (FDR) based on the procedure of Benjamini and Hochberg [21]. The *q*-values may be interpreted as follows: if only genes with a *q*-value < 0.05 are selected as significant, then the FDR for the experiment will be less than 0.05.

In a linear regression of MammU6 C_T_ values on RNA concentration, most of the variance in C_T_ value was explained by RNA concentration (R^2^ = 0.98). We tried adding the UV dose to the linear regression equation, but it was not statistically significant (*p*-value = 0.502). Thus there is no evidence that ssUVR influences MammU6 expression.

All data, both processed and raw, has been deposited in GEO (www.ncbi.nlm.nih.gov/geo) (accession number GSE75621).

## Results

### The expression of UV-responsive miRNAs in the melanocytes of melanoma patients is mainly down-regulated

**Fig 1:**
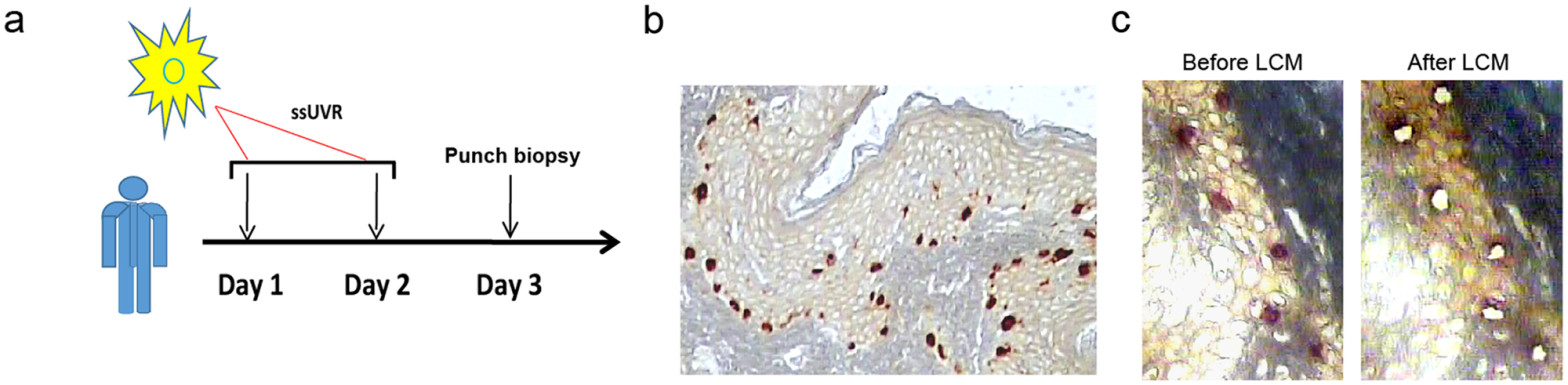
Capture of melanocytes from the epidermis of human skin after exposure to ssUVR. **(a)** UV-irradiation scheme. On day one and two, the posterior shoulder of each volunteer received an ssUVR dose (6 mm circle) of 4X their baseline MED (see Methods). On day three, a punch biopsy removed the irradiated site, as well as an adjacent un-irradiated site. **(b)** Immunohistochemical staining of human skin. Antibody to MART-1 (Melan-A) was used for identifying melanocytes present in the epidermis of skin. Brownish red cells indicate the melanocytes. (c) Stained slides before and after LCM. A pure population of melanocytes was captured by using the Arcturus XTTM LCM system.

In an effort to uncover new UVR-mediated responses in melanocytes while residing within their natural morphological microenvironment, we performed with ethical approval a photobiological procedure on 17 fair-skinned females between the ages of 31 to 46. Nine of these females were melanoma patients (see S1 Table) with a history of having only one primary melanoma, while the other eight females had no history of skin cancer. Each of the individuals in these two groups were importantly tested for their sensitivity to ssUVR (see Methods) by determining the lowest dose of irradiation at which sunburn would occur on their skin (MED). Once this value had been established, a dose of ssUVR roughly equivalent to that which would have been gained on unprotected skin if one were to spend ~ 5 h at a beach was used to irradiate a very small area on the posterior shoulder of every volunteer (see Methods). After 24 h, this area was again exposed to the same amount of UV irradiation (Fig 1a). Skin biopsies of the irradiated site along with an adjacent non-exposed site were then taken 24 h after the second UV irradiation with the expectation of identifying only those UV-responsive miRNAs that had not fully returned to their original expression level.

We used an antibody (Melan-A) against a melanocyte-associated antigen (MART-1) to rapidly identify and subsequently capture by LCM the irradiated or un-irradiated melanocytes from these tissue samples (Fig 1b and 1c, and S1a Fig). Since this antibody did not detect the MART-1 antigen within macrophages (S1b Fig), it was unlikely that these cells, as well as other cell types, were being inadvertently captured by LCM. Additional proof for the purity of the isolated melanocytes is seen by qRT-PCR on RNAs extracted from melanocytes or undifferentiated keratinocytes selected by LCM from the basal cell layer (the deepest part) of the epidermis.

In this analysis, we found that the RNA of the irradiated melanocytes could express the melanocyte ‘lineage-specific’ transcription factor MITF but not the epidermal markers cytokeratins 10 and 14. Both of these are normally expressed in keratinocytes, but not in melanocytes (S2a and S2b Fig) [22, 23]. That only cytokeratin 14 was expressed in the RNA of the isolated keratinocytes was not surprising (S2b Fig), given that the expression of cytokeratin 10 can only occur in keratinocytes located well above the basal cell layer [23]. Taken together, these results unambiguously show that a relatively pure population of irradiated or un-irradiated melanocytes can be isolated from the tissue of human skin under our experimental conditions.

**Fig 2:**
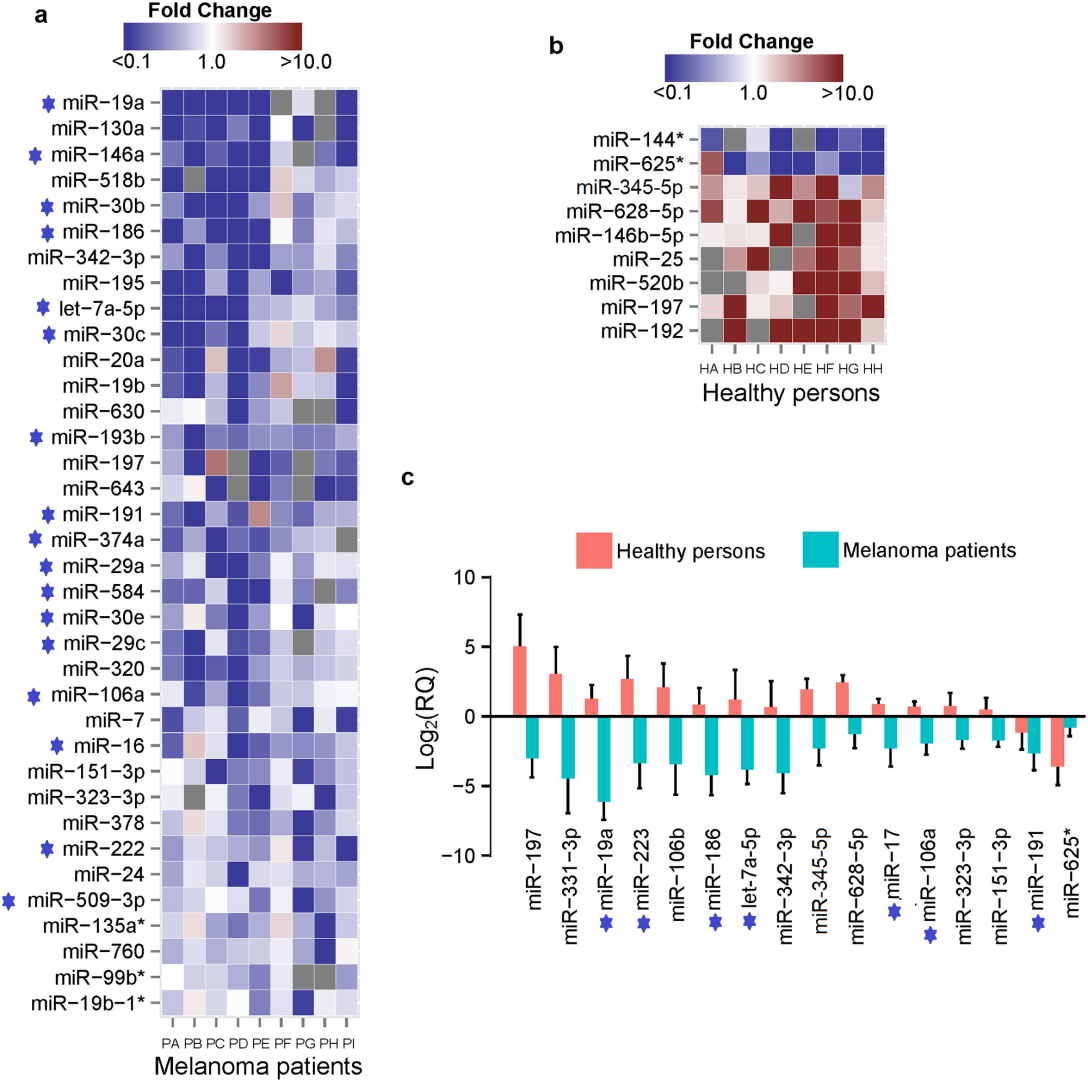
MicroRNAs differentially expressed in melanocytes of skin after exposure to ssUVR. **(a and b)** A color-coded heat map representations of UV-miRNAs in melanocytes commonly expressed in melanoma patients or healthy individuals. The map in **(a)** ranks each miRNA by their fold-change (RQ), beginning with those having the most negative RQ value (top) to the highest negative RQ value (bottom), whereas the map in **(b)** ranks each from the most negative to the most positive RQ value. The color bars at the top of **(a)** and **(b)** represent the normalized intensity of down-regulated or up-regulated UV-miRNAs with the shaded blue corresponding to a decrease in expression and that of shaded red to an increase in expression. The left margin shows the representative miRNAs and the bottom of the columns show each individual sample. MicroRNAs in the heat map of **(a)** and which match those previously identified in melanoma tissue are marked by a star. **(c)** An identical set of UV-miRNAs in the melanocytes of patients or healthy persons is effected differently by ssUVR. The *y*-axis indicates relative gene expression, and the whisker bars indicate the standard error of the mean. MicroRNAs that match those previously identified in melanoma tissue are marked by a star.

Our next priority was to look for differences in the expression of miRNAs between the irradiated and un-irradiated melanocytes within the skin of human volunteers. In this investigation, we considered procedures that complement the technique of LCM, and this entailed the use of TaqMan microRNA array cards (A and B) in-conjunction with qRT-PCR. Together, these cards consist of 667 assays and multiple endogenous control genes for correcting differences in the levels of RNA between samples. The control gene that was selected for all our studies was MammU6, a highly abundant (~400,000 copies/cell) and stably expressed non-coding small nuclear RNA (snRNA) whose synthesis has been shown to be unaffected by UVB [24], and more importantly, to the combined effect of solar simulated UVA + UVB as well (Fig 1a and S3 Fig). We began by comparing the miRNA profiles of irradiated versus un-irradiated melanocytes isolated from the skin of each healthy women by LCM. On average, 122 out of 667 miRNAs were deregulated with a fold-change > or < than 1.5 in the irradiated melanocytes, with a great majority of them being up-regulation in expression (data not shown). This result was in striking contrast to that which was observed in the irradiated melanocytes of women with a history of melanoma in that a much higher proportion of the deregulated miRNAs, which on average were ~133 across all samples, were down-regulated in expression (data not shown). We then identified UV-miRNAs that were differentially expressed between the irradiated and un-irradiated melanocytes in each of the healthy persons and the melanoma patients. In this analysis, we identified 36 UV-miRNAs whose levels of concentrations had significantly decreased in the irradiated melanocytes of all nine of the melanoma patients (Fig 2a). Among these, as marked in Fig 2a, are the presence of 17 miRNAs [e.g., miR-186 (*P*<0.007), let-7a-5p (*P*<0.003), miR-29a (*P*<0.003), miR-342-3p (P<0.003), and miR-30c (P<0.011)] that correspond to those whose expression was reported to be reduced or diminished in excised melanoma tissue [25–27]. Although there were several UV- responsive miRNAs that were commonly up-regulated in the melanocytes in seven out of nine melanoma patients (data not shown), we chose not to report them since they did not meet a strong enough statistical significance to pass our threshold. Finally, our analysis identified nine UV-miRNAs that were commonly deregulated in the melanocytes of the eight healthy women (Fig 2b). Seven of these were up-regulated in expression [e.g., miR-628-5p (*P*<0.007), miR-146b-5p (*P*<0.015), miR-197 (*P*<0.015), and miR-192 (*P*<0.031)], while the other two were significantly down-regulated in expression, namely miR-144* (*P*<0.031) and miR-625* (*P*<0.039). A listing of all the UV-miRNAs, including their relative expression levels (fold-change) as well as their *p*- and *q*-values (i.e. adjusting for multiple testing), is given in S2 Table. Overall, these results indicate that miRNAs in the melanocytes of melanoma patients respond differently to ssUVR than those in the melanocytes of healthy persons.

We then questioned whether individual UV-miRNAs in the melanocytes of either the melanoma patients or healthy persons was being effected differently by ssUVR. By using a Mann-Whitney test, we identified sixteen miRNAs whose levels were reduced in the melanocytes of the melanoma patients, but surprisingly increased in those of the healthy persons (Fig 2c and S4 Fig). Seven of these miRNAs (e.g., miR-19a, miR-223, miR-17, and miR191) are identical to those that have been found to be repressed in melanoma tissue [26]. This result suggests that the melanocytes of melanoma patients respond differently to ssUVR than those of healthy persons and that their effected UV-miRNAs could be associated with the early development of melanoma.

**Fig 3:**
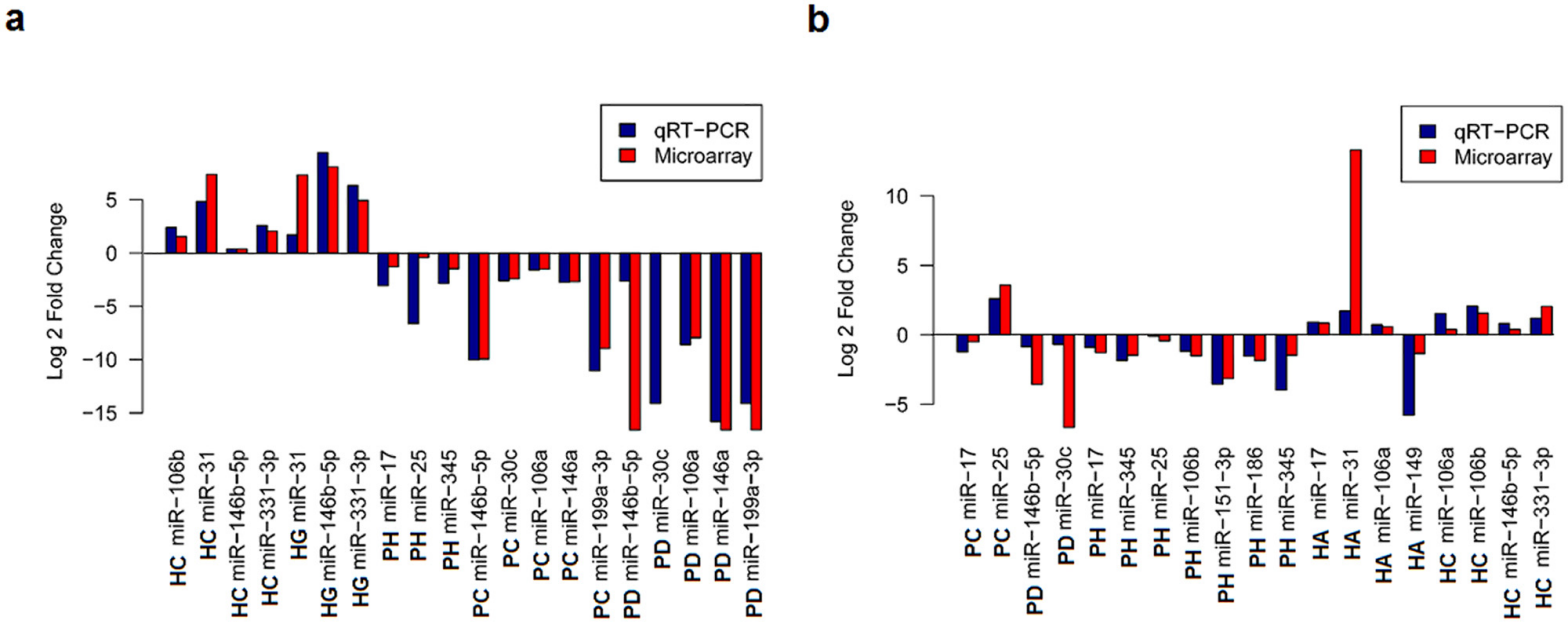
Verification of the expression levels of UV-miRNAs by qRT-PCR. **(a)** The human microRNA array cards were validated by performing qRT-PCR analysis on the same pre-amplified RNA (see Methods) that was used on these cards. Pre-amplified RNAs corresponding to the irradiated melanocytes of healthy persons C and G (**HC** and **HG**) or melanoma patients C, D and H (**PC**, **PD**, and **PH**) were chosen at random for monitoring the expression of relevant UV-miRNAs. In all cases, the direction of the fold change is the same for both qRT-PCR and Microarray. Fishers exact test on values dichotomized as up/down regulated yields a *p*-value = 1.29E-05. **(b)** The expression levels of twelve UV-miRNAs were further validated by conducting qRT-PCR analysis on RNA without pre-amplification and extracted from irradiated melanocytes newly derived by LCM from the samples of healthy persons A and C (**HA** and **HC**) or melanoma patients C, D, and H (**PC**, **PD**, and **PH**). Fishers exact test in this panel yields a *p*-value = 1.323E-05.

Two approaches were used to validate the expression levels of the UV-miRNAs observed in our studies. We first performed individual TaqMan qPCR on the same amplified RNA samples (see Methods) that were used in the microarray cards, and which were derived from the irradiated and un-irradiated melanocytes of both melanoma patients and healthy persons. As shown in Fig 3a, the results obtained by the qPCR assays were in concordance to those obtained from the microarrays (*P* < 1.29E-05), thereby indicating the reliability of using these cards with only limited amounts of RNA. We then did individual TaqMan qPCR assays on non-amplified RNAs newly isolated from irradiated and un-irradiated melanocytes derived by LCM from the same tissue samples (both healthy persons and patients) used in the profiling. The tissue samples in this analysis were randomly chosen, as were the groups of eight up-regulated and eleven down-regulated miRNAs. Again, these assays (*P* < 1.32E-05) revealed good concordance with similar trends in the expression patterns of UV-miRNAs when compared to the microarray results (Fig 3b). Overall, these results critically show the feasibility of profiling UV-miRNAs in melanocytes following their capture by LCM.

### UV-miRNA-based regulatory networks identify novel functions in melanocytes prone to melanoma

**Fig 4:**
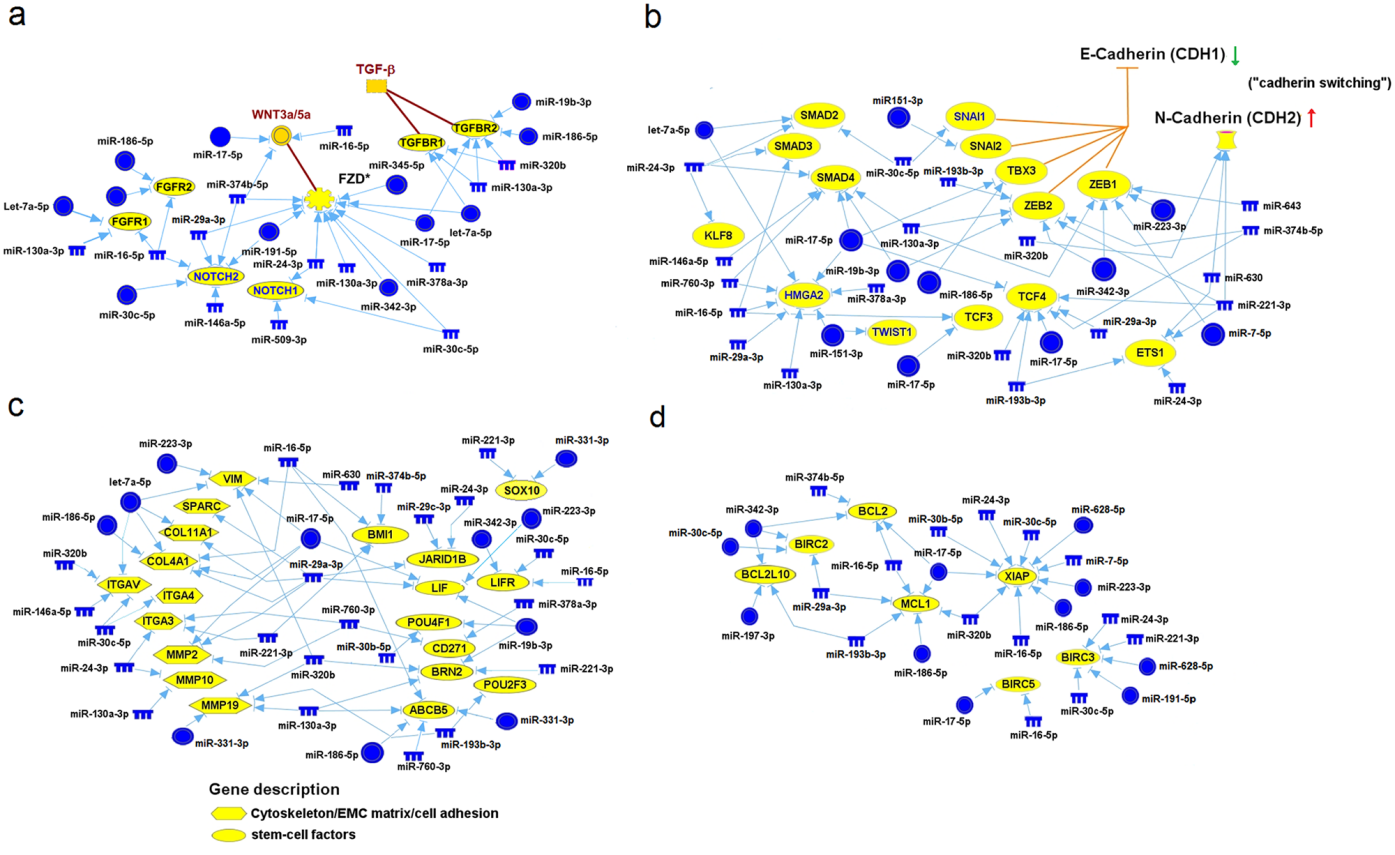
Four interlocking regulatory networks showing statistical relationships between a subset of repressed UV-miRNAs and EMT signature genes. Highlighted in networks **a** and **b** is a hypothetical scheme illustrating the modulation of EMT signaling by TGF-β and/or WNT3a/5a, and the shift from E-cadherin to N-cadherin expression (“cadherin switching”). These events are fundamental to the EMT. A potential gain-of-function in genes within networks **c** and **d** are respectfully associated with cells acquiring stem-cell like properties and resistance to apoptosis, additional hallmarks of the EMT. FZD* represents three of the WNT receptor genes (Frizzled), FZD2, FZD5, and FZD7. The blue colored notches and circles represent the down-regulated UV-miRNAs listed in Fig 2a and 2c, respectively, while the yellow colored circles signify the potentially up-regulated genes.

To understand the functions of the repressed UV-miRNAs in possibly contributing to the causality of aberrant processes in melanocytes prone to melanoma, we used DIANA-TarBase, which holds a collection of experimentally supported miRNA-gene interactions [19], and Ingenuity’s miRNA Target Filter function (see Methods). The Ingenuity program uses experimental observations and prediction algorithms for unbiased identification of miRNA-target genes, and one of these considers the total context-score, as defined by TargetScan [20]. We began by interrogating the datasets of UV-miRNAs associated with the melanocytes of melanoma patients (Fig 2a) and the melanocytes of healthy persons versus melanoma patients (Fig 2c). This led to the construction of a number of UV-miRNA-gene regulatory networks and a listing of parameters that describes the targeting efficiency of the UV-miRNAs in each of these (S3 Table). Strikingly, four of these networks, which are interlinked, were indicative of EMT-like activities (Fig 4), a result consistent with findings linking EMT-like processes with melanoma progression and the phenotypic heterogeneity of melanoma cells as well [28–30].

The first EMT-like network has eighteen different repressed UV-miRNAs (Fig 4a), which are connected to genes that are intrinsic to EMT-signaling pathways and known for their activities in responding to extracellular cues [31]. Of significance are the melanoma-related genes NOTCH1, NOTCH2, WNT3a, and WNT5a, each of which are associated with two or more UV-miRNAs that can directly target these respective genes (see S3 Table). Other members of this network include the frizzled genes (e.g.FZD3 and FZD5), which serve as receptors in WNT signaling, and the FGF and TGF-β receptors, FGFR1/2 and TGFBR1/2 (Fig 4a) [31]. Of note is the large number of down-regulated UV-miRNAs that are connected to the melanoma gene NOTCH2 (Fig 4a). Among these are four different UV-miRNAs (miR-16-5p/16, miR-29a-3p/29a, miR-191-5p/191, and miR-30c-5p/30c), which have been experimentally verified in regulating this gene (S3 Table). Also of note, is that two of these UV-miRNAs (miR-191-5p and miR-30c-5p) were found to be up-regulated in the irradiated melanocytes of healthy persons (Fig 2b and 2c).

The second EMT-like network consists of twenty-one different UV-miRNAs (Fig 4b), and prominently intersects with major EMT-inducing transcription factors (EMT-TF’s) such as ZEB (ZEB1/ZEB2), TWIST, SNAIL (SNAIL1/SNAIL2), and TBX3 [31], all of which are well-known transcriptional repressors [31]. The repressor TBX3 is of special importance since it has been shown to be up-regulated in melanoma, and a key contributor to the formation of melanoma [32, 33]. Other transcriptional factors such as KLF8, TCF3, TCF4, and SMAD are also included in this network, and each of them are known to be involved in EMT processes as well [31]. What is also quite evident in this network is the association of a large number of repressed UV-miRNAs that have been experimentally predicted (S3 Table) in targeting the so-called “master genes” of EMT, namely ZEB1 and ZEB2 [31]. Since the effect of more than one miRNA on a gene target is summative [8], a gain-of-function in these two genes is highly expected in the melanocytes of patients. Notably, one of the UV-miRNAs (miR-342-3p) which converges on both ZEB1 and ZEB2 in this network (Fig 4b) is up-regulated in the irradiated melanocytes of healthy individuals (Fig 2c). Fig 4b also contains the chromatin remodeler HMGA2, a gene that is known to be overexpressed in primary melanoma and up-regulated by TGF-β during EMT [31, 34]. HMGA2 is coupled to eight different UV-miRNAs, and seven of these have been experimentally verified in their ability to target this gene directly (S3 Table). Also in this set are three matching UV-miRNAs (miR-151-3p, miR-17-5p/17, and miR-25) which are statistically up-regulated in the irradiated melanocytes of healthy individuals (Fig 2b and 2c). Perhaps the most important aspect of this regulatory network, however, is that it also includes a system known as “cadherin switching,” which is noted as a hallmark of EMT and has relevancy to melanoma [29–31, 35]. This switch involves the down-regulation of E-cadherin (CDH1), the gatekeeper of EMT, and concurrently, the up-regulation of N-cadherin (CDH2), a molecule vital to the EMT process [31]. We predict that CDH2 will display a gain-of-function due to its connection to three repressed UV-miRNAs in the melanocytes of patients (Figs 2a and 4b), with two of these being of a high targeting efficacy, while the other (miR-221-3p) has been experimentally shown to target CDH2 directly (S3 Table). It is also highly probable that the function of CDH1 in these cells will be potentially blocked by the transcriptional repressors SNAIL, TBX3, and ZEB1/2 (Fig 4b), given that each of them is being potentially released from inhibition due to their association with a combined total of fifteen down-regulated UV-miRNAs (Fig 2a and 2c).

The process of EMT also involves the loss of cell-cell contact, the remodeling of the extracellular matrix, and usually, the acquisition of stem cell-like properties [31]. Known genes that participate in these processes are included in the third EMT-like regulatory network (Fig 4c), and each were identified by their statistical relationship with one or more of the down-regulated UV-miRNAs in the melanocytes of patients (Fig 2a and 2c). Those that effect a change in cell-cell cohesiveness (e.g. VIM, SPARC, COL4A1, and the integrins, ITGA4 and ITGAV), and have been shown to be over expressed in human or mouse melanoma samples [3, 29, 36, 37], are each associated with one or more UV-miRNAs (Fig 4c and S3 Table). The ability of ITGAV to regulate cancer progression and locally activate TGF-β/SMAD EMT-signaling pathways [38], is likely the result of the two UV-miRNAs miR-25 and miR-192, which can target this gene directly [19], and are up-regulated in the irradiated melanocytes of healthy persons (Fig 2b). Genes (e.g. BRN2, POU2F3, and SOX10) expressed in melanoblasts, and those (e.g. JARID1B, CD271, and ABCB5) which help to endow melanoma cells with stem-like properties and cellular plasticity [39], are also in this network (Fig 4c). A gain-of-function is predicted for JARID1B and CD271 due to their association with two or more UV-miRNAs (Fig 4c and S3 Table). A gain-of-function in the melanoma stem cell marker ABCB5 is also probable because of its connection with five down-regulated UV-miRNAs (Fig 4c and S3 Table), and two of them (miR-186-5p/186 and miR-331-3p) are identical to the ones that are up-regulated in the irradiated melanocytes of healthy persons (Fig 2c). Finally, this network also shows four repressed UV-miRNAs converging onto BRN2/POU3F2 and two onto POU4F1 (Fig 4c). What is interesting about these two genes, both of which are overexpressed in melanoma cell lines, is that an up-regulated BRN2 can participate in the de-differentiation of human melanocytes, and POU4F1 appears to have a critical role in melanoma development [40].

When EMT processes are linked to the progression of cancer, the cells involved often acquire a resistance to apoptosis [41], and primary melanomas exemplify this by over expressing a number of anti-apoptotic genes[42], an occurrence that is most likely a function of EMT-TFs [43]. In accordance, our fourth EMT-like regulatory network contains sixteen repressed UV-miRNAs and seven interconnecting apoptotic resistant or pro-survival genes (Fig 4d), all of which can modulate melanoma cell survival [42]. Two of them (MCL1, XIAP) encode a member of the Bcl-2 family and an potent apoptotic inhibitor [42]. Remarkably, MCL1 and XIAP are connected to a combined total of sixteen UV-miRNAs, four of which are shared by both of these genes (Fig 4d). In addition, fourteen of these have been experimentally validated (S3 Table), and four match UV-miRNAs up-regulated in the irradiated melanocytes of healthy persons (Fig 2c). Interestingly, miR-192-5p/192 is the most highly elevated (~5-fold) UV-miRNA in these cells (Fig 2b), and is known for its ability to target XIAP directly [19].

Overall, these results strongly indicate that in response to ssUVR, the melanocytes of melanoma patients in contrast to those of healthy individual undergo a switch to an EMT-like phenotype, a process mediated by the occurrence of down-regulated UV-miRNAs. By contrast, melanocytes from healthy individuals switch to a protective mode after UV-irradiation, as judged by the many up-regulated UV-miRNAs.

**Fig 5:**
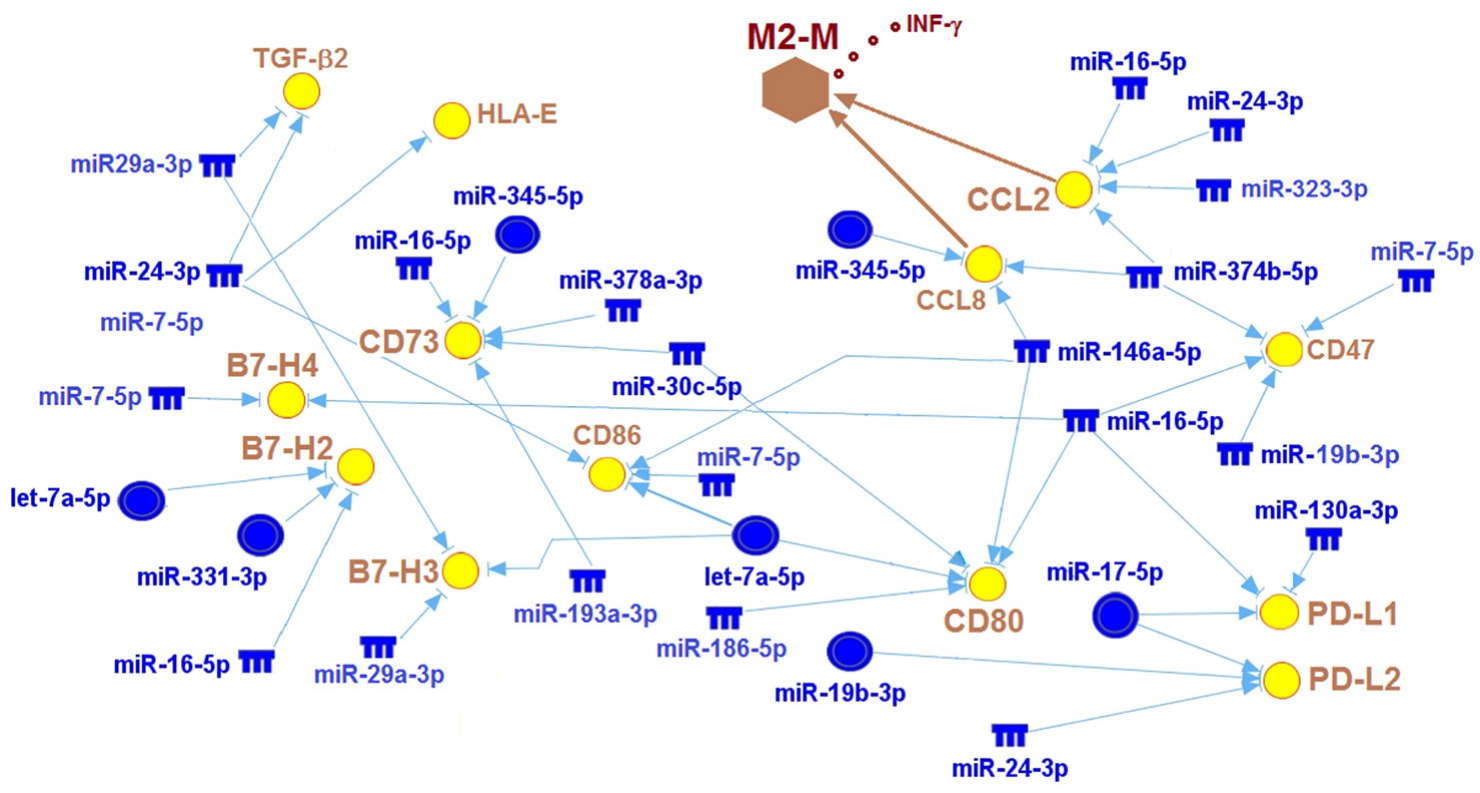
A regulatory network showing statistical relationships between 14 repressed UV-miRNAs and 13 immuno-evasive signature genes. Superimposed on the network is a hypothetical mechanism resulting from a potential gain-of-function in CCL2 and CCL8, both of which are known to attract M2-type macrophages (M2-M), and in turn the release of INF-γ, a cytokine reportedly involved in up-regulating critical immuno-evasive genes such as PD-L1, PD-L2, and B7-H2. The blue colored notches and circles represent the down-regulated UV-miRNAs listed in Fig 2a and 2c, respectively, while the yellow colored circles signify the potentially up-regulated genes.

Melanomas use several strategies in order to escape immune-mediated destruction. One involves the release of immunosuppressive factors (e.g. CCL2 and TGF-β2), while another is to express checkpoint ligands such as PD-L1/L2, B7-H, and CD73, which can then react with receptors on cytotoxic T-lymphocytes (CTLs), thereby damping their activity [44]. We therefore determined by IPA analysis (see Methods) whether any of the down-regulated UV-miRNAs in the melanocytes of patients (Fig 2a and 2c) could generate a gene regulatory network for immune-suppression. Notably, a network consisting of linkages between these miRNAs and genes that block the host immune response was produced (Fig 5). In this network genes such as CD73 and CD80, which can transmit inhibitory signals to T cells [44], and the ligands CCL2 and CCL8, which bind to receptors on T cells and/or M2-type macrophages and thereby suppress their attack [44, 45], are individually associated with three or more UV-miRNAs (Fig 5 and S3 Table). Interestingly, microRNAs miR-345-5p and miR-146a-5p, which are up-regulated in the irradiated melanocytes of healthy persons (Fig 2b and 2c), have been experimentally verified as targeting CD73 and CD80, respectively [19]. A potential gain-of-function in CCL2 and CCL8 is interesting, given that these ligands are induced by UVB in the melanocytes of HGF/SF-transgenic mice, which are prone to UV-induced malignant transformation [6]. This induction then leads to the recruitment of macrophages to neonatal skin with the release of IFN-γ, which in turn helps to promote immuno-evasion [6]. Finally, and perhaps more importantly is that the functional outputs of this network also include the melanoma signature genes PD-L1 and PD-L2 (Fig 5) [44, 45]. Both are expressed in melanoma tissues [45], and there is evidence to suggest that PD-L1 can limit T cell activity [44]. As shown in Fig 5, these two ligands are individually associated with three different repressed UV-miRNAs, and amongst them are miR-17-5p/17 and miR-19b-3p/19a, which match those that are up-regulated in the irradiated melanocytes of healthy persons (Fig 2c). Both of these UV-miRNAs have been experimentally verified in targeting PD-L1 directly (S3 Table). Overall, the integrative analysis shown here strongly indicates that some form of immunosuppression could be prematurely occurring in the irradiated melanocytes of patients as opposed to those of healthy persons.

## Discussion

In this study, we show that physiological doses of simulated sunlight can perturb the balance of miRNA expression in human melanocytes while in their natural environment. However, the most surprising observation from our study was that this effect differs dramatically between the melanocytes of melanoma patients and healthy persons, with UV-miRNAs being predominantly down-regulated in the former. We believe then that the UV-miRNA signature observed in the melanocytes of these patients represents a key first step in the subsequent conversion of these cells into a new cell state, thereby rendering these individuals prone to melanoma. This is supported by the power of network-based analysis which took into account only those genes that had been experimentally validated in being jointly regulated by more than one specific miRNA [19]. This approach enabled us to identify a number of UV-miRNA regulatory modules containing genes with a gain-of-function and having activities that are known to be operational in pathways dedicated to the processes of EMT and immune-evasion. Although these two programs have been implicated in the invasion-metastasis cascade of melanoma [46], they have yet to be linked to the effects of solar UVR in the early stage of melanoma development. Clinically, this new discovery could be potentially useful in having new prognostic markers and/or therapeutic treatments for a particular type of melanoma patient.

In addition to its role in helping cells escape from a primary tumor, the induction of EMT also correlates with numerous other properties such as a tumor-initiating cell phenotype, a resistance to apoptosis, and classically, the loss of E-cadherin (CDH1), which subsequently gives cells, including melanoma, the ability to migrate [31, 47]. The acquisition of an EMT phenotype in melanoma has been shown to be associated with signaling pathways such as Wnt/β-catenin, FGF, Notch, and TGF-β, and the transcriptional repressors SNAIL, TBX3, TWIST, and ZEB [48]. Our findings indicate that through miRNA down-regulation the functional activities of these pathways and transcriptional factors are no longer inhibited in the melanocytes of melanoma patients in response to ssUVR. Remarkably, our findings also show that the melanocytes of healthy individuals upon exposure to ssUVR do not reflect these phenotypic changes. EMT is also a de-differentiation process accompanied by molecular events that drive cells to acquire stem-cell like properties [49]. The findings presented here describe a number of melanoma stem cell markers (e.g. ABCB5, JARDIB, and CD271) [39] that are linked to UV-miRNAs in the irradiated melanocytes of patients. The potential of conferring a partial “stemness” to melanoma prone melanocytes due to the dynamics of acquiring a gain-of-function in these particular genes could in fact correspond to the early beginnings of a self-renewing state, a phenotype that is not found in the irradiated melanocytes of healthy persons. In effect, this could give rise to a type of melanocyte with an unlimited potential for undergoing cell divisions with long periodicities, and thereby accumulate mutations as required for malignant transformation.

The identification of a UV-miRNA-regulated network that inter-connects with a number of de-repressed genes (e.g. PD-L1 and CCL2) involved in immune evasion in the melanocytes of melanoma patients was unexpected, and to the best of our knowledge, the first evidence of its kind. What this finding suggests is that these cells hold the potential of evading immune-mediated elimination at the onset of their transformation, thereby making possible the accumulation of both genetic and epigenetic changes before evolving into a melanoma. UVB-mediated alterations in immune functions is not without precedent in that short term exposure of mutant melanocytes in a melanoma mouse model results in the up-regulation of a variety of immune evasive genes as a function of IFN-γ production [6].

Finally, our data support the concept that persons who are susceptible to melanoma have melanocytes that may harbor a defective miRNA biogenesis program. Because of this, these cells have the potential to acquire cancer promoting phenotypes in response to environmental pressures such as solar UVR, and therefore, a greater probability of developing into an early stage melanoma. In fact, abnormalities in the biogenesis and processing of miRNAs have been reported in cutaneous melanoma, and alterations in the concentration of the miRNA-processing enzyme DICER, as well as genetic and epigenetic changes at the chromosomal level, have been implicated in this dysfunction [50]. What remains to be determined, however, is exactly how solar UVR directly or indirectly effects an aberrant miRNA system in human melanocytes, and especially while in their natural microenvironment. Knowledge such as this would undoubtedly provide an additional approach to melanoma therapy.

## Supporting Information

S1 Fig Capture of pure melanocytes by LCM. **(a)** A human tissue section was stained with the Melan-A antibody, and afterwards, the melanocytes were captured by LCM and visualized on the LCM cap (x 40). **(b)** Human tissue sections cut in sequence (serial) were stained with the Melan-A or the CD68 antibody (x 20). Sections were derived from a biopsy sample after irradiation. Black arrows show MART-1 labeled material and its absence within a serial section stained with anti-CD68.
(TIF)

S2 Fig Purity of LCM-derived melanocytes. **(a)** Approximately 200 irradiated melanocytes or keratinocytes were captured from an irradiated tissue section by LCM. Expression of cytokeratins 10 and 14 (specific to keratinocytes) or MITF and MART-1 (specific to melanocytes) was determined by qPCR. RNA samples were analyzed in triplicate with the 18S rRNA gene serving as an endogenous control. The average ΔC_T_ values (C_T mRNA_—C_T 18S rRNA_) corresponding to the genes of interest was calculated. **(b)** Bar plots indicate quantitative differences in the expression of MITF, MART-1 and the cytokeratins 10 (K10) and 14 (K14) between the melanocytes and keratinocytes after normalization. The letters M and K signify melanocytes and keratinocytes, respectively. *K10; the absence of K10 expression in LCM-derived keratinocytes is consistent with the finding that it is only expressed in keratinocytes located well above the basal cell layer [23].
(TIF)

S3 Fig The in expression of MammU6 cultured melanocytes is un-affected by ssUVR. Human melanocytes were made quiescent (see Methods), exposed to ssUVR, and then returned to complete medium for 24 h. At that time, the cells were again exposed to ssUVR, placed in complete medium, and then harvested 24 h later for the extraction of RNA, followed by qRT-PCR analysis. **(a)** Correlation of RNA input to the threshold of cycle (C_T_) values for the expression of MammU6 at various doses of ssUVR. **(b)** The expression levels of *XPC* and *GADD45A* in cultured melanocytes after exposure to ssUVR (see Methods). The results depicted in the graph are consistent with the findings of others. The values in the Y-axis are based on the ΔΔC_T_ method. Error bars indicate standard error of the mean. The * and ** are *P*-values < 0.015 and 0.020, respectively.
(TIF)

S4 Fig Heat map showing identical miRNAs in melanocytes that respond differently to ssUVR in melanoma patients vs. healthy persons. The rows and columns represent each of the miRNAs and the individual samples, respectively. Table (right panel) lists the respective miRNAs and their associated *Q*-value.
(PNG)

S1 Table Clinical characteristics of melanoma patients. (DOCX)

S2 Table Listing of UV-responsive miRNAs commonly expressed in the melanocytes of melanoma patients or healthy persons. (DOCX)

S3 Table Significantly repressed UV-miRNAs and their experimental and predicted.interactions with genes regulating EMT and immuno-evasion. (DOCX)
