# Supplementary figures and images for: The Response of microRNAs to Solar UVR in Skin-Resident Melanocytes Differs between Melanoma Patients and Healthy Persons
Source: PLoS One. 2016 May 5;11(5):e0154915. doi: 10.1371/journal.pone.0154915 (PMC4858311; doi:10.1371/journal.pone.0154915)

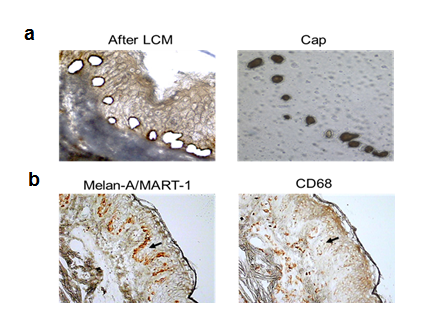

Supplement: S1 Fig — (a) A human tissue section was stained with the Melan-A antibody, and afterwards, the melanocytes were captured by LCM and visualized on the LCM cap (x 40). (b) Human tissue sections cut in sequence (serial) were stained with the Melan-A or the CD68 antibody (x 20). Sections were derived from a biopsy sample after irradiation. Black arrows show MART-1 labeled material and its absence within a serial section stained with anti-CD68. (TIF) [file pone.0154915.s001.tif]

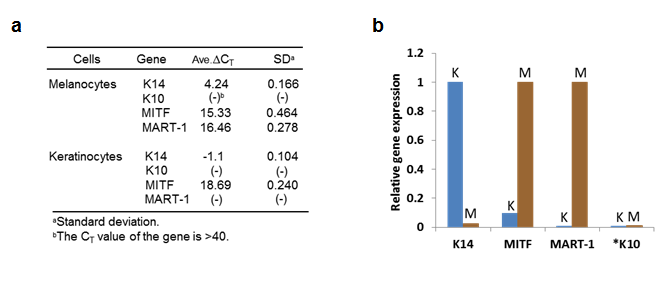

Supplement: S2 Fig — (a) Approximately 200 irradiated melanocytes or keratinocytes were captured from an irradiated tissue section by LCM. Expression of cytokeratins 10 and 14 (specific to keratinocytes) or MITF and MART-1 (specific to melanocytes) was determined by qPCR. RNA samples were analyzed in triplicate with the 18S rRNA gene serving as an endogenous control. The average ΔCT values (CT mRNA—CT 18S rRNA) corresponding to the genes of interest was calculated. (b) Bar plots indicate quantitative differences in the expression of MITF, MART-1 and the cytokeratins 10 (K10) and 14 (K14) between the melanocytes and keratinocytes after normalization. The letters M and K signify melanocytes and keratinocytes, respectively. *K10; the absence of K10 expression in LCM-derived keratinocytes is consistent with the finding that it is only expressed in keratinocytes located well above the basal cell layer [23]. (TIF) [file pone.0154915.s002.tif]

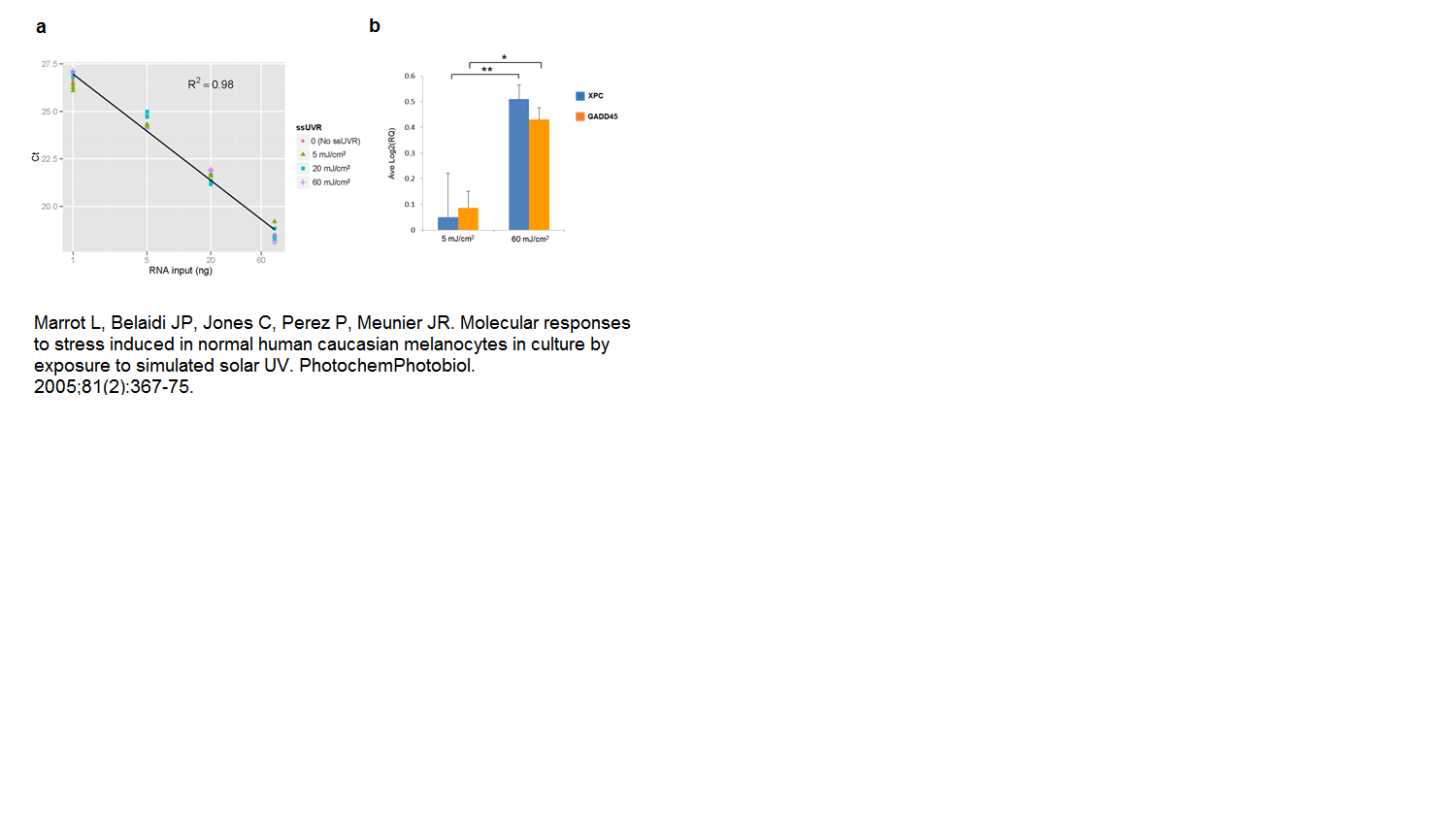

Supplement: S3 Fig — Human melanocytes were made quiescent (see Methods), exposed to ssUVR, and then returned to complete medium for 24 h. At that time, the cells were again exposed to ssUVR, placed in complete medium, and then harvested 24 h later for the extraction of RNA, followed by qRT-PCR analysis. (a) Correlation of RNA input to the threshold of cycle (CT) values for the expression of MammU6 at various doses of ssUVR. (b) The expression levels of XPC and GADD45A in cultured melanocytes after exposure to ssUVR (see Methods). The results depicted in the graph are consistent with the findings of others. The values in the Y-axis are based on the ΔΔCT method. Error bars indicate standard error of the mean. The * and ** are P-values < 0.015 and 0.020, respectively. (TIF) [file pone.0154915.s003.tif]

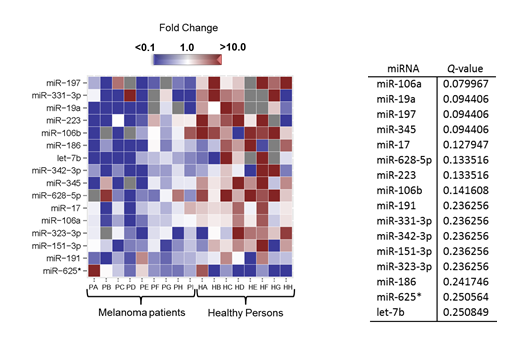

Supplement: S4 Fig — The rows and columns represent each of the miRNAs and the individual samples, respectively. Table (right panel) lists the respective miRNAs and their associated Q-value. (PNG) [file pone.0154915.s004.png]
