# Supplementary material for: The Response of microRNAs to Solar UVR in Skin-Resident Melanocytes Differs between Melanoma Patients and Healthy Persons
Source: PLoS One. 2016 May 5;11(5):e0154915. doi: 10.1371/journal.pone.0154915 (PMC4858311; doi:10.1371/journal.pone.0154915)
Supplement: S1 Table — (DOCX) [file pone.0154915.s005.docx]

**S1 Table. Clinical Characteristics of Melanoma Patients.**

| Sample ID | Sex | Age | Type | Breslow (mm) | Clark Level | SNB |
| --- | --- | --- | --- | --- | --- | --- |
| A | F | 43 | SSN | 0.30 | 111 | Negative |
| B | F | 40 | Nodular | 1.50 | 1V | Negative |
| C | F | 44 | In situ | - | 1 | Negative |
| D | F | 38 | SSM | 1.18 | 1V | Negative |
| E | F | 38 | SSM | 0.59 | 11 | Negative |
| F | F | 38 | SSM | 0.49 | 11 | Negative |
| G | F | 42 | SSM | 0.27 | 11 | Negative |
| H | F | 46 | SSM | 0.84 | 1V | Negative |
| I | F | 42 | SSM | 0.72 | 1V | Negative |

All samples are primary malignant melanoma. SSM, Superficial spreading

Melanoma; SNB, sentinel node status.
