# Supplementary material for: The Response of microRNAs to Solar UVR in Skin-Resident Melanocytes Differs between Melanoma Patients and Healthy Persons
Source: PLoS One. 2016 May 5;11(5):e0154915. doi: 10.1371/journal.pone.0154915 (PMC4858311; doi:10.1371/journal.pone.0154915)
Supplement: S2 Table — (DOCX) [file pone.0154915.s006.docx]

**Supplementary Table 2. Listing of UV-responsive miRNAs commonly**

**expressed in the melanocytes of melanoma patients or healthy persons.**

**Mature miRNA ID^a^** **Log2 (RQ)** ***P*-value** ***Q***-**value**

*Melanoma Patients*

| let-7a-5p | -3.815038228 | 0.00390625 | 0.03671875 |
| --- | --- | --- | --- |
| hsa-miR-106a | -1.934635738 | 0.00390625 | 0.03671875 |
| hsa-miR-193b | -3.084606156 | 0.00390625 | 0.03671875 |
| hsa-miR-195 | -3.877491789 | 0.00390625 | 0.03671875 |
| hsa-miR-24 | -1.296862693 | 0.00390625 | 0.03671875 |
| hsa-miR-29a | -2.356417277 | 0.00390625 | 0.03671875 |
| hsa-miR-320 | -1.977035252 | 0.00390625 | 0.03671875 |
| hsa-miR-342-3p | -4.069366411 | 0.00390625 | 0.03671875 |
| hsa-miR-151-3p | -1.735492571 | 0.00390625 | 0.03671875 |
| hsa-miR-7 | -1.927819396 | 0.00390625 | 0.03671875 |
| hsa-miR-186 | -4.209255060 | 0.0078125 | 0.03671875 |
| hsa-miR-222 | -1.564972520 | 0.0078125 | 0.03671875 |
| hsa-miR-509-3p | -1.259031342 | 0.0078125 | 0.03671875 |
| hsa-miR-760 | -1.157511339 | 0.0078125 | 0.03671875 |
| hsa-miR-130a | -5.860729221 | 0.0078125 | 0.03671875 |
| hsa-miR-146a | -5.268770738 | 0.0078125 | 0.03671875 |
| hsa-miR-29c | -2.004778439 | 0.0078125 | 0.03671875 |
| hsa-miR-323-3p | -1.677406509 | 0.0078125 | 0.03671875 |
| hsa-miR-374a | -2.565950613 | 0.0078125 | 0.03671875 |
| hsa-miR-584 | -2.203361806 | 0.0078125 | 0.03671875 |
| hsa-miR-16 | -1.827090093 | 0.01171875 | 0.045898438 |
| hsa-miR-30b | -4.472546422 | 0.01171875 | 0.045898438 |
| hsa-miR-30c | -3.704581119 | 0.01171875 | 0.045898438 |
| hsa-miR-378 | -1.656755783 | 0.01171875 | 0.045898438 |
| hsa-miR-19a | -6.113187629 | 0.015625 | 0.056490385 |
| hsa-miR-99b* | -1.004566457 | 0.015625 | 0.056490385 |
| hsa-miR-135a* | -1.157533503 | 0.01953125 | 0.06330819 |
| hsa-miR-19b-1* | -0.893482869 | 0.01953125 | 0.06330819 |
| hsa-miR-30e | -2.198424432 | 0.01953125 | 0.06330819 |
| hsa-miR-518b | -4.953623800 | 0.0234375 | 0.0734375 |
| hsa-miR-191 | -2.650510722 | 0.02734375 | 0.080322266 |
| hsa-miR-19b | -3.246057467 | 0.02734375 | 0.080322266 |
| hsa-miR-630 | -3.100285903 | 0.03125 | 0.086397059 |
| hsa-miR-643 | -2.690391886 | 0.03125 | 0.086397059 |
| hsa-miR-20a | -3.665310222 | 0.0390625 | 0.104910714 |
| hsa-miR-197 | -3.010042686 | 0.046875 | 0.122395833 |

*Healthy persons*

| miR-628-5p | 2.441084403 | 0.0078125 | 0.4453125 |
| --- | --- | --- | --- |
| miR-146b-5p | 3.739265291 | 0.015625 | 0.4453125 |
| miR-197 | 5.020505871 | 0.015625 | 0.4453125 |
| miR-192 | 5.83550820 | 0.03125 | 0.4453125 |
| miR-25 | 3.863864363 | 0.03125 | 0.4453125 |
| miR-520b | 4.236394882 | 0.03125 | 0.4453125 |
| miR-144* | -5.098572967 | 0.03125 | 0.4453125 |
| miR-345 | 1.93507936 | 0.0390625 | 0.4453125 |
| miR-625* | -3.606935163 | 0.0390625 | 0.4453125 |

^a^miRNAs are ranked by *P*- and *Q*-value
