# Supplementary material for: The Response of microRNAs to Solar UVR in Skin-Resident Melanocytes Differs between Melanoma Patients and Healthy Persons
Source: PLoS One. 2016 May 5;11(5):e0154915. doi: 10.1371/journal.pone.0154915 (PMC4858311; doi:10.1371/journal.pone.0154915)
Supplement: S3 Table — (DOCX) [file pone.0154915.s007.docx]

| Supplementary Table 3. Significantly repressed UV-miRNAs and their experimental and predicted interactions with genes regulating EMT and immuno-evasion. | | | | |
| --- | --- | --- | --- | --- |
| Gene symbol | **miRNA** | **Aggregate PCT** | ***Total context + score** | **Evidence (ref.)** |
|  | | | | |
| *EMT-like regulatory networks* | |  |  |  |
| *a* |  |  |  |  |
| WNT3a/5 | **miR-16-5p** | **-** | **-** | **validated (9)** |
|  | **miR-374b-5p** | **-** | **-** | **validated (9)** |
|  | **miR-17-5p** | **-** | **-** | **validated (9)** |
|  | **miR-193b-3p** | **-** | **-** | **validated (9)** |
|  |  |  |  |  |
| TGFBR1 | **Let-7a-5p** | **-** | **-** | **validated (9)** |
|  | **miR-130a-3p** | **0.75** | **-0.164** | **predicted (10)** |
|  | **miR-320b** | **-** | **-0.02** | **predicted (10)** |
|  |  |  |  |  |
| TGFBR2 | **miR-17-5p** | **-** | **-** | **validated (9)** |
|  | **miR-19b-3p** | **-** | **-** | **validated (9)** |
|  | **miR-130a-3p** | **-** | **-** | **validated (9)** |
|  | **miR-186-5p** | **-** | **-** | **validated (9)** |
|  | **miR-320b** | **-** | **-0.018** | **predicted (10)** |
|  |  |  |  |  |
| FGFR2 | **miR-186-5p** | **-** | **-** | **validated (9)** |
| FGFR1 | **miR-16-5p** | **-** | **-** | **validated (9)** |
|  | **miR-130a-3p** | **-** | **-** | **validated (9)** |
|  | **Let-7a-5p** | **-** | **-** | **validated (9)** |
|  |  |  |  |  |
| NOTCH2 | **miR-16-5p** | **-** | **-** | **validated (9)** |
|  | **miR-29a-3p** | **-** | **-** | **validated (9)** |
|  | **miR-191-5p** | **-** | **-** | **validated (9)** |
|  | **miR-30c-5p** | **-** | **-** | **validated (9)** |
|  | **miR-146a-5p** | **0.225** | **-0.106** | **predicted (10)** |
|  | **mir-374b-5p** | **-** | **-0.044** | **predicted (10)** |
|  |  |  |  |  |
| NOTCH1 | **miR-24-3p** | **-** | **-** | **validated (9)** |
|  | **miR-509-3p** | **-** | **-0.231** | **predicted (10)** |
|  | **miR-30c-5p** | **-** | **-** | **validated (9)** |
|  |  |  |  |  |
| FZD5 | **miR-191-5p** | **0.438** | **-0.198** | **predicted (10)** |
|  | **mir-374b-5p** | **-** | **-0.118** | **predicted (10)** |
|  | **miR-378a-3p** | **-** | **-0.42** | **predicted (10)** |
|  | **miR-24-3p** | **-** | **-** | **validated (9)** |
|  | **miR-29a-3p** | **-** | **-** | **validated (9)** |
|  | **miR-130a-3p** | **-** | **-** | **validated (9)** |
|  |  |  |  |  |
| FZD2 | **Let-7a-5p** | **-** | **-** | **validated (9)** |
|  | **miR-24-3p** | **-** | **-0.218** | **predicted (10)** |
|  | **miR-30c-5p** | **0.749** | **-0.088** | **predicted (10)** |
|  | **miR-342-3p** | **-** | **-0.31** | **predicted (10)** |
|  |  |  |  |  |
| FZD7 | **miR-17-5p** | **0.506** | **-0.066** | **predicted (10)** |
|  |  |  |  |  |
| *b* |  |  |  |  |
| SMAD2 | **miR-30c-5p** | **-** | **-** | **validated (9)** |
|  | **miR-24-3p** | **-** | **-** | **validated (9)** |
|  | **miR-17-5p** | **-** | **-** | **validated (9)** |
|  | **Let-7a-5p** | **0.902** | **-0.086** | **predicted (10)** |
|  |  |  |  |  |
| SMAD3 | **miR-16-5p** | **-** | **-** | **validated (9)** |
|  | **miR-24-3p** | **-** | **-** | **validated (9)** |
|  |  |  |  |  |
| SMAD4 | **miR-17-5p** | **-** | **-** | **validated (9)** |
|  | **miR-19b-3p** | **-** | **-** | **validated (9)** |
|  | **miR-760-3p** | **-** | **-0.345** | **predicted(10)** |
|  | **miR-146a-5p** | **-** | **-** | **validated (9)** |
|  | **miR-24-3p** | **-** | **-** | **validated (IPA)**** |
|  | **miR-130a-3p** | **-** | **-** | **validated (9)** |
|  |  |  |  |  |
| KLF8 | **miR-24-3p** | **-** | **-0.384** | **predicted (10)** |
|  |  |  |  |  |
| HMGA2 | **miR-17-5p** | **-** | **-** | **validated (9)** |
|  | **miR-19b-3p** | **-** | **-** | **validated (9)** |
|  | **miR-151-3p** | **-** | **-** | **validated (9)** |
|  | **miR-16-5p** | **-** | **-** | **validated (9)** |
|  | **miR-130a-3p** | **-** | **-** | **validated (9)** |
|  | **miR-378a-3p** | **-** | **-0.207** | **predicted (10)** |
|  | **Let-7a-5p** | **-** | **-** | **validated (9)** |
|  | **miR-29a-3p** | **-** | **-** | **validated (9)** |
|  |  |  |  |  |
| TWIST1 | **miR-151-3p** | **-** | **-0.223** | **predicted (10)** |
|  |  |  |  |  |
| TCF3 | **miR-16-5p** | **-** | **-** | **validated (9)** |
|  | **miR-17-5p** | **-** | **-** | **validated (9)** |
|  |  |  |  |  |
| TCF4 | **miR-193b-3p** | **-** | **-** | **validated (9)** |
|  | **miR-320b** | **-** | **-** | **validated (9)** |
|  | **miR-17-5p** | **-** | **-** | **validated (9)** |
|  | **miR-374b-5p** | **-** | **-** | **validated (9)** |
|  | **miR-29a-3p** | **-** | **-** | **validated (9)** |
|  | **miR-221-3p** | **-** | **-** | **validated (9)** |
|  |  |  |  |  |
| ETS1 | **miR-24-3p** | **-** | **-** | **validated (9)** |
|  | **miR-221-3p** | **0.122** | **-0.278** | **predicted (10)** |
|  | **miR-630** | **-** | **-0.209** | **predicted (10)** |
|  | **miR-193b-3p** | **-** | **-** | **validated (IPA)** |
|  |  |  |  |  |
| SNAI1 | **miR-30c-5p** | **-** | **-** | **validated (9)** |
|  |  |  |  |  |
| SNAI2 | **miR-151-3p** | **-** | **-** | **validated (9)** |
|  |  |  |  |  |
| TBX3 | **miR-186-5p** | **-** | **-** | **validated (9)** |
|  | **miR-17-5p** | **-** | **-** | **validated (9)** |
|  |  |  |  |  |
| ZEB1 | **miR-130a-3p** | **0.716** | **-0.131** | **predicted (10)** |
|  | **miR-7-5p** | **-** | **-** | **validated (9)** |
|  | **miR-342-3p** | **-** | **-0.109** | **predicted (10)** |
|  | **miR-643** | **-** | **-0.253** | **predicted (10)** |
|  | **miR-223-3p** | **0.349** | **-0.047** | **predicted (10)** |
|  |  |  |  |  |
| ZEB2 | **miR-221-3p** | **-** | **-** | **validated (9)** |
|  | **miR-374b-5p** | **-** | **-** | **validated (9)** |
|  | **miR-130a-3p** | **0.684** | **-0.234** | **predicted (10)** |
|  | **miR-342-3p** | **-** | **-0.06** | **predicted (10)** |
|  | **miR-19b-3p** | **0.304** | **-0.03** | **predicted (10)** |
|  | **miR-193b-3p** | **-** | **-** | **validated (9)** |
|  |  |  |  |  |
| CDH2 | **miR-221-3p** | **-** | **-** | **validated (9)** |
|  | **miR-630** | **-** | **-0.209** | **predicted (10)** |
|  | **miR-320b** | **-** | **-0.153** | **predicted (10)** |
|  |  |  |  |  |
| *c* |  |  |  |  |
| VIM | **miR-630** | **-** | **-0.212** | **predicted (10)** |
|  | **miR-320b** | **-** | **-** | **validated (9)** |
|  | **miR-17-5p** | **-** | **-** | **validated (9)** |
|  | **Let-7a-5p** | **-** | **-** | **validated (IPA)** |
|  | **miR-223-3p** | **-** | **-** | **validated (IPA)** |
|  |  |  |  |  |
| BMI1 | **miR-374b-5p** | **-** | **-** | **validated (9)** |
|  | **miR-221-3p** | **-** | **-** | **validated (9)** |
|  | **miR-16-5p** | **-** | **-** | **validated (9)** |
|  |  |  |  |  |
| SPARC | **miR-29a-3p** | **-** | **-** | **validated (9)** |
|  |  |  |  |  |
| COL11A1 | **miR-29a-3p** | **-** | **-0.329** | **predicted (10)** |
|  | **Let-7a-5p** | **0.772** | **-0.098** | **predicted (10)** |
|  |  |  |  |  |
| COL4A1 | **Let-7a-5p** | **-** | **-** | **validated (9)** |
|  | **miR-29a-3p** | **-** | **-** | **validated (9)** |
|  | **miR-186-5p** | **-** | **-** | **validated (9)** |
|  | **miR-17-5p** | **-** | **-** | **validated (9)** |
|  | **miR-16-5p** | **-** | **-** | **Validated (9)** |
|  |  |  |  |  |
| ITGAV | **Let-7a-5p** | **-** | **-** | **validated (9)** |
|  | **miR-146a-5p** | **0.103** | **-0.103** | **predicted (10)** |
|  | **miR-320b** | **-** | **-** | **validated (9)** |
|  | **miR-30c-5p** | **-** | **-** | **validated (9)** |
|  |  |  |  |  |
| ITGA4 | **miR-30c-5p** | **-** | **-** | **validated (9)** |
|  |  |  |  |  |
| ITGA3 | **miR-24-3p** | **0.38** | **-0.093** | **predicted (10)** |
|  | **miR-221-3p** | **0.305** | **-0.102** | **predicted (10)** |
|  | **miR-760-3p** | **-** | **-0.401** | **predicted (10)** |
|  |  |  |  |  |
| MMP2 | **miR-17-5p** | **-** | **-** | **validated (9)** |
|  | **miR-29a-3p** | **-** | **-** | **validated (9)** |
|  | **miR-760-3p** | **-** | **-0.534** | **predicted (10)** |
|  |  |  |  |  |
| MMP10 | **miR-24-3p** | **-** | **-0.207** | **predicted (10)** |
|  | **miR-130a-3p** | **-** | **-0.275** | **predicted (10)** |
|  |  |  |  |  |
| MMP19 | **miR-320b** | **-** | **-0.02** | **predicted (10)** |
|  | **miR-193b-3p** | **0.258** | **-0.451** | **predicted (10)** |
|  | **miR-130a-3p** | **-** | **-0.263** | **predicted (10)** |
|  | **miR-331-3p** | **-** | **-0.399** | **predicted (10)** |
|  |  |  |  |  |
| SOX10 | **miR-331-3p** | **-** | **-0.418** | **predicted (10)** |
|  | **miR-221-3p** | **0.122** | **-0.231** | **predicted (10)** |
|  |  |  |  |  |
| JARID1B | **miR-29a-3p** | **-** | **-** | **validated (9)** |
|  | **miR-29c-3p** | **-** | **-** | **validated (9)** |
|  | **miR-24-3p** | **0.208** | **-0.55** | **predicted (10)** |
|  |  |  |  |  |
| LIF | **miR-29a-3p** | **-** | **-** | **validated (9)** |
|  | **miR-17-5p** | **0.515** | **-0.03** | **predicted (10)** |
|  | **miR-223-3p** | **0.359** | **-0.168** | **predicted (10)** |
|  | **miR-19b-3p** | **-** | **-** | **validated (9)** |
|  |  |  |  |  |
| LIFR | **miR-16-5p** | **-** | **-** | **validated (9)** |
|  | **miR-30c-5p** | **-** | **-** | **validated (9)** |
|  | **miR-342-3p** | **-** | **-0.059** | **predicted (10)** |
|  |  |  |  |  |
| POU4F1/BRN3a | **miR-30b-5p** | **-** | **-** | **validated (9)** |
|  | **miR-19b-3p** | **0.876** | **-0.093** | **predicted (10)** |
|  |  |  |  |  |
| NGFR/CD271 | **miR-378a-3p** | **-** | **-0.249** | **predicted (10)** |
|  | **miR-760-3p** | **-** | **-0.281** | **predicted (10)** |
|  |  |  |  |  |
| POU3F2/BRN2 | **miR-221-3p** | **-** | **-** | **validated (9)** |
|  | **miR-130a-3p** | **-** | **-** | **validated (9)** |
|  | **miR-19b-3p** | **0.383** | **-0.181** | **predicted (10)** |
|  | **miR-320b** | **-** | **-0.09** | **predicted (10)** |
|  |  |  |  |  |
| ABCB5 | **miR-186-5p** | **-** | **-** | **validated (9)** |
|  | **miR-16-5p** | **-** | **-0.385** | **predicted (10)** |
|  | **miR-130a-3p** | **-** | **-0.36** | **predicted (10)** |
|  | **miR-331-3p** | **-** | **-0.338** | **predicted (10)** |
|  | **miR-760-3p** | **-** | **-0.208** | **predicted (10)** |
|  |  |  |  |  |
| POU2F3 | **miR-193b-3p** | **-** | **-0.3** | **predicted (10)** |
|  |  |  |  |  |
| *d* |  |  |  |  |
| BCL2 | **miR-374b-5p** | **-** | **-** | **validated (9)** |
|  | **miR-17-5p** | **-** | **-** | **validated (9)** |
|  | **miR-16-5p** | **-** | **-** | **validated (9)** |
|  | **miR-342-3p** | **-** | **-0.426** | **predicted (10)** |
|  |  |  |  |  |
| BIRC2 | **miR-30c-5p** | **-** | **-** | **validated (9)** |
|  | **miR-342-3p** | **-** | **-0.13** | **predicted (10)** |
|  | **miR-29b-3p** | **0.023** | **-0.318** | **predicted (10)** |
|  |  |  |  |  |
| BCL2L10 | **miR-197-3p** | **-** | **-0.201** | **predicted (10)** |
|  | **miR-193b-3p** | **0.076** | **-0.325** | **predicted (10)** |
|  | **miR-342-3p** | **-** | **-0.538** | **predicted (10)** |
|  |  |  |  |  |
| MCL1 | **miR-186-5p** | **-** | **-** | **validated (9)** |
|  | **miR-193b-3p** | **-** | **-** | **validated (9)** |
|  | **miR-16-5p** | **-** | **-** | **validated (9)** |
|  | **miR-17-5p** | **-** | **-** | **validated (9)** |
|  | **miR-320b** | **-** | **-** | **validated (9)** |
|  | **miR-29a-3p** | **-** | **-** | **validated (9)** |
|  |  |  |  |  |
| XIAP | **miR-17-5p** | **-** | **-** | **validated (9)** |
|  | **miR-30b-5p** | **-** | **-** | **validated (9)** |
|  | **miR-24-3p** | **0.318** | **-0.323** | **predicted (10)** |
|  | **miR-30c-5p** | **-** | **-** | **validated (9)** |
|  | **miR-628-5p** | **-** | **-** | **validated (9)** |
|  | **miR-7-5p** | **-** | **-** | **validated (9)** |
|  | **miR-223-3p** | **0.39** | **-0.289** | **predicted (10)** |
|  | **miR-186-5p** | **-** | **-** | **validated (9)** |
|  | **miR-16-5p** | **-** | **-** | **validated (9)** |
|  | **miR-320b** | **-** | **-** | **validated (9)** |
|  |  |  |  |  |
| BIRC5 | **miR-16-5p** | **-** | **-** | **validated (9)** |
|  | **miR-17-5p** | **-** | **-** | **validated (9)** |
|  |  |  |  |  |
| BIRC3 | **miR-24-3p** | **-** | **-** | **validated (9)** |
|  | **miR-30c-5p** | **-** | **-** | **validated (9)** |
|  | **miR-221-3p** | **-** | **-** | **validated (9)** |
|  | **miR-191-5p** | **-** | **-** | **validated (9)** |
|  | **miR-628-5p** | **-** | **-** | **validated (9)** |
|  |  |  |  |  |
|  |  |  |  |  |
| *Immuno-evasion-like regulatory network* | | |  |  |
|  |  |  |  |  |
| CD73/NT5E | **miR-378a-3p** | **-** | **-0.312** | **predicted (10)** |
|  | **miR-30c-5p** | **-** | **-** | **validated (IPA)** |
|  | **miR-345-5p** | **-** | **-** | **validated (9)** |
|  | **miR-16-5p** | **-** | **-** | **validated (9)** |
|  | **miR-193a-3p** | **0.355** | **-0.592** | **predicted (10)** |
|  |  |  |  |  |
| B7-H4/VTCN1 | **miR-16-5p** | **-** | **-** | **validated (9)** |
|  | **miR-7-5p** | **-** | **-** | **validated (9)** |
|  |  |  |  |  |
| B7-H2/ICOSLG | **miR-16-5p** | **-** | **-** | **validated (9)** |
|  | **Let-7a-5p** | **-** | **-** | **validated (9)** |
|  | **miR-331-3p** | **-** | **-0.676** | **predicted (10)** |
|  |  |  |  |  |
| B7-H3/CD276 | **miR-29a-3p** | **-** | **-** | **validated (9)** |
|  |  |  |  |  |
| CD86 | **miR-24-3p** | **-** | **-0.355** | **predicted (10)** |
|  | **Let-7a-5p** | **0.597** | **-0.204** | **predicted (10)** |
|  | **miR-7-5p** | **-** | **-** | **validated (9)** |
|  | **miR-146a-5p** | **-** | **-** | **validated (9)** |
|  |  |  |  |  |
| HLA-E | **miR-24-3p** | **-** | **-** | **validated (9)** |
|  |  |  |  |  |
| TGF-β2 | **miR-24-3p** | **-** | **-0.313** | **predicted (10)** |
|  | **miR-29a-3p** | **-** | **-** | **validated (9)** |
|  | **miR-29c-3p** | **-** | **-** | **validated (9)** |
|  |  |  |  |  |
| CD80 | **Let-7a-5p** | **-** | **-** | **validated (9)** |
|  | **miR-30c-5p** | **-** | **-0.221** | **predicted (10)** |
|  | **miR-146a-5p** | **0.84** | **-0.295** | **predicted (10)** |
|  | **miR-186-5p** | **-** | **-** | **validated (9)** |
|  | **miR-16-5p** | **0.255** | **-0.734** | **predicted (10)** |
|  |  |  |  |  |
| CCL8 | **miR-146a-5p** | **-** | **-** | **validated (9)** |
|  | **miR-345-5p** | **-** | **-** | **predicted (10)** |
|  | **miR-374b-5p** | **-** | **-0.137** | **predicted (10)** |
|  |  |  |  |  |
| CCL2 | **miR-16-5p** | **-** | **-** | **validated (9)** |
|  | **miR-323-3p** | **-** | **-** | **validated (9)** |
|  | **miR-374b-5p** | **-** | **-0.162** | **predicted (10)** |
|  | **miR-24-3p** | **-** | **-** | **validated (9)** |
|  |  |  |  |  |
| CD47 | **miR-19b-3p** | **-** | **-** | **validated (9)** |
|  | **miR-16-5p** | **-** | **-0.466** | **predicted (10)** |
|  | **miR-37b-5p** | **-** | **-** | **validated (9)** |
|  | **miR-7-5p** | **-** | **-** | **validated (9)** |
|  |  |  |  |  |
| PD-L1/CD274 | **miR-17-5p** | **-** | **-** | **validated (9)** |
|  | **miR-16-5p** | **-** | **-** | **validated (9)** |
|  | **miR-130a-3p** | **-** | **-** | **validated (9)** |
|  |  |  |  |  |
| PD-L2/PDCD1LG2 | **miR-24-3p** | **-** | **-** | **validated (9)** |
|  | **miR-17-5p** | **0.759** | **-0.695** | **predicted (10)** |
|  | **miR-19b-3p** | **0.143** | **-0.402** | **predicted (10)** |
